# HEP-Net opinion on the management of ascites and its complications in the setting of decompensated cirrhosis in the resource constrained environment of Pakistan

**DOI:** 10.12669/pjms.36.5.2407

**Published:** 2020

**Authors:** Bushra Ali, Adnan Salim, Altaf Alam, Bader Faiyaz Zuberi, Zeeshan Ali, Zahid Azam, Lubna Kamani, Javed Iqbal Farooqi, Muhammed Salih, Arif Amir Nawaz, Asad Ali Chaudhry, Zahid Yasin Hashmi, Masood Siddique

**Affiliations:** 1 Bushra Ali, Fatima Memorial Medical and Dental College, Lahore, Pakistan; 2 Adnan Salim, Shaikh Zayed Postgraduate Medical Institute, Lahore, Pakistan; 3 Altaf Alam, Shaikh Zayed Postgraduate Medical Institute, Lahore, Pakistan; 4 Bader Faiyaz Zuberi, Dow Medical College, Dow University of Health Sciences, Karachi, Pakistan; 5 Zeeshan Ali, Jinnah Postgraduate Medical Centre, Karachi, Pakistan; 6 Zahid Azam, NILGID, Dow University of Health Sciences, Karachi, Pakistan; 7 Lubna Kamani, Liaquat National Hospital, Karachi, Pakistan; 8 Javed Iqbal Farooqi, Lady Reading Hospital, Peshawar, Pakistan; 9 Muhammed Salih, Quaid e Azam International Hospital, Islamabad, Pakistan; 10 Arif Amir Nawaz, Fatima Memorial Medical and Dental College, Lahore, Pakistan; 11 Asad Ali Chaudhry, Gujranwala Liver Centre Gujranwala, Pakistan; 12 Zahid Yasin Hashmi, Liver Center, Faisalabad, Pakistan; 13 Masood Siddique, Jinnah Memorial Hospital, Rawalpindi, Pakistan

**Keywords:** Cirrhosis, Ascites, Portal Hypertension, Spontaneous bacterial peritonitis, Hepatorenal syndrome, refractory ascites;

## Abstract

Approximately one half of patients develop ascites within 10 years of diagnosis of compensated cirrhosis. It is a poor prognostic indicator, with only 50% surviving beyond two years. Mortality worsens significantly to 20% to 50% at one year if the ascites becomes refractory to medical therapy. Pakistan has one of the highest prevalence of viral hepatitis in the world and patients with ascites secondary to liver cirrhosis make a major percentage of both inpatient and outpatient burden. Studies indicate that over 80% of patients admitted with ascites have liver cirrhosis as the cause.

This expert opinion suggests proper assessment of patients with ascites in the presence of underlying cirrhosis. This expert opinion includes appropriate diagnosis and management of uncomplicated ascites, refractory ascites and complicated ascites (including spontaneous bacterial peritonitis (SBP) ascites, hepatorenal syndrome (HRS) and hyponatremia. The purpose behind this expert opinion is to help consultants, postgraduate trainees, medical officers and primary care physicians optimally manage their patients with cirrhosis and ascites in a resource constrained setting as is often encountered in a developing country like Pakistan.

## Burden of Disease

Approximately one half of patients develop ascites within 10 years of diagnosis of compensated cirrhosis.^1^ It harbingers a new phase of cirrhosis called decompensated cirrhosis resulting in significant deterioration of prognosis. It is a poor prognostic indicator, with only 50% surviving beyond two years.^2^,^3^ Mortality worsens significantly to 20% to 50% at one year if the ascites becomes refractory to medical therapy.^4^ Pakistan has one of the highest prevalence of viral hepatitis in the world and patients with ascites secondary to liver cirrhosis make a major percentage of both inpatient and outpatient burden. Studies indicate that over 80% of patients admitted with ascites have liver cirrhosis as the cause.^5^-^8^ Most common cause of cirrhosis in Pakistan is reported as hepatitis C infection in over half of the such patients.^9^

## Diagnosis of Ascites

The initial evaluation of ascites includes detailed history, physical examination, abdominal ultrasound, liver function tests including serum albumin and INR, renal function tests, serum electrolytes and spot urinary electrolytes. Ascitic fluid should be analyzed to calculate serum albumin to ascitic fluid albumin gradient (SAAG).^10^ A SAAG of more than or equal to 1.1 is defined as high SAAG ascites while it is low SAAG if this ratio is less than 1.1. The following document elaborates upon management of high SAAG ascites only.

**Table:** GRADING SYSTEM USED IN RECOMMENDATIONS

DESCRIPTION		SYMBOL
*GRADING OF EVIDENCE*		
High	Further research is very unlikely to change our confidence in the estimate of effect	A
Moderate	Further research is likely to have an important impact on our confidence in the estimate of effect and may change the estimate	B
Low or very low	Further research is very likely to have an important impact on our confidence in the estimate of effect and is likely to change the estimate. Any estimate of effect is uncertain	C
*GRADING RECOMMENDATION*		
Strong recommendation	Factors influencing the strength of the recommendation included the quality of evidence, presumed patient-important outcomes, and cost	I
Weaker recommendation	Variability in preferences and values, or more uncertainty: more likely a weak recommendation is warranted Recommendation is made with less certainty: higher cost or resource consumption	II

## DEFINITIONS

### Uncomplicated ascites

Uncomplicated ascites is defined as ascites that is not infected and is not associated with refractoriness to conventional medical treatment, development of hyponatremia, spontaneous bacterial peritonitis, or hepatorenal syndrome.^10^

### Complicated ascites

Complicated ascites is defined as ascites with complications of one or more of the following: Spontaneous bacterial peritonitis (SBP) hepatorenal syndrome (HRS) and hyponatremia.^10^

### Refractory ascites

Ascites that cannot be mobilized with maximum dose of diuretics (furosemide 160 mg/day and spironolactone 400 mg/day) or the early recurrence (within 2 weeks) of which [i.e., after Large volume paracentesis (LVP) of 5 liters or more of ascitic fluid] cannot be satisfactorily prevented by medical therapy.^11^,^12^

### Management of Uncomplicated Ascites

Ascites may be classified into three grades depending upon the quantity of free fluid in the peritoneal cavity. Choice of treatment depends upon this clinical quantification of fluid:^13^,^14^

It is essential to carry out biochemical, microscopic and microbiologic analysis of ascitic fluid. Biochemistry includes total protein, albumin, LDH and glucose content of ascitic fluid. The total leucocyte count and differential leucocyte count (TLC and DLC) must be checked. Ascitic fluid ought to be sent in blood culture bottles for culture/sensitivity. Reagent strips for leucocyte esterase have been successfully used for bedside diagnosis of SBP in some studies.^15^

It is important to rule out spontaneous bacterial peritonitis (SBP) if ascites in new in onset or worsening of ascites is noted. SBP should also be ruled out in those who get hospitalized with any complication of cirrhosis or for any other medical or surgical reason.^16^,^17^ The total protein content will also help assess the future risk of SBP. Ascitic fluid analysis should still be carried out at every admission regardless of the time period between current admission and previous discharge to rule out SBP.

### Recommendations

- All patients with ascites must undergo a complete history, examination and relevant investigations along with ascitic fluid analysis to calculate SAAG and to rule out spontaneous bacterial peritonitis

**Table:** 

Grades of ascites	Definition	Treatment
Grade 1	Mild ascites only detectable on ultrasound	No treatment
Grade 2	Moderate ascites with moderate symmetrical distension of abdomen	Salt restriction and diuretics
Grade 3	Large or gross ascites with marked abdominal distension/tense ascites	Therapeutic paracentesis followed by salt restriction and diuretics
- Ascitic fluid analysis is essential in cases of cirrhosis with new onset ascites, worsening ascites, or those admitted with other complications of cirrhosis.

### Management of moderate ascites

Treatment of moderate ascites is aimed at counteracting the increased renal reabsorption of sodium by restricting salt intake and increasing sodium excretion with diuretics.

## DIURETICS AND SALT RESTRICTION

A diet with sodium content of less than 2g or 88mmol per day (equivalent to 5g of table salt per day) must be stressed upon. This is roughly equal to one heaped teaspoon or two flat teaspoons of table salt. Diuretic dose can be increased stepwise every week, while maintaining a ratio of 40mg of furosemide for every 100mg of spironolactone. A maximum daily weight loss of 0.5kg/day is reasonable in the absence of pedal edema. There is no restriction to weight loss if massive pedal edema accompanies ascites.^1^,^4^ Mild to moderate ascites should be managed by modest salt restriction and diuretic therapy with spironolactone or an equivalent in the first instance. Diuretics should be added in a stepwise fashion while maintaining sodium restriction. Gross ascites should be treated with therapeutic paracentesis followed by colloid volume expansion, and diuretic therapy. Refractory ascites is managed by repeated large volume paracentesis or insertion of a transjugular intrahepatic portosystemic stent shunt (TIPS).^18^

Diuretics may require dose adjustment in summer months due to increased fluid losses. Diligent follow up of patients, every 1-2 weeks to begin with, is required as the dose of diuretics is stepped up for weight loss documentation, orthostatic hypotension, electrolyte imbalance, pre-renal azotemia, and signs of portosystemic encephalopathy as a result of diuretic overdose.^19^

All patients with ascites should not be advised to restrict their fluid intake. Restriction of fluid intake should only be advised in the small minority who have documented serum sodium of below 125 mmol/L.^14^,^20^ Intravenous furosemide is best avoided since it causes rapid fluid shifts and an acute reduction in renal perfusion leading to azotemia. This azotemia may be mistaken for hepatorenal syndrome when in fact it is diuretic induced and is corrected once diuretics are withheld and dehydration is corrected.^19^

Spot urinary sodium to potassium ratio is a convenient alternative to 24-hour urinary sodium while assessing outpatient response to diuretic therapy. When this ratio is more than one, it suggests adequate diuresis.^19^ Adequate diuresis is also suggested by a urinary sodium excretion of more than 30 mmol/day.^19^ Regular albumin infusions as per need or every two weeks have been recommended in some studies as an adjunct to diuretics and improved survival has been claimed.^4^,^10^,^12^ It is recommended that diuretics be taken in once daily dose in morning for better compliance and response.^21^

### Recommendations

- Patients should be advised not to take more than 5 grams of table salt or 88 mmol/2g of sodium daily. (I-A)
- Diuretics should be given in a once daily dose (I-A)
- Diuretics should be introduced in a step-up dose at a ratio of 100:40 of spironolactone: furosemide(I-A)
- Weight, blood pressure, serum electrolytes, creatinine must be checked at every OPD visit along with examination for orthostatic hypotension and signs of portosystemic encephalopathy. (I-A)
- The frequency of outpatient visit should be once every one to four weeks depending upon the response to treatment(I-A)
- Spot urinary sodium to potassium ratio can be checked to assess response to diuresis. (II-A)
- Patients should be advised to stop diuretics if urinary sodium is < 30 mmol/day. (II-A)
- Diuretics should also be stopped if serum sodium is < 120 mmol/L (1-B)
- Regular use of intravenous furosemide should be best avoided. (I-B)
- Fluid restriction to 1000 ml/day is effective in increasing serum sodium concentration in only a minority of patients with hypervolemic hyponatremia, but may be effective in preventing a further reduction in serum sodium levels (1-A).

### Effects of Other Drugs Commonly Used By The Cirrhotic Patient

- Studies have shown that beta-blockers may increase mortality in patients with refractory ascites by causing hemodynamic instability.^22^ Hence they should be used with caution (II-A).
- Angiotensin converting enzyme inhibitors and angiotensin receptor blockers are best avoided in patients with cirrhosis and ascites.^23^,^24^ (II-B).
- It is best not to prescribe aminoglycosides, NSAIDs and other nephrotoxic drugs in patients with cirrhosis and ascites and should be used when other antibiotic or analgesics options are not available and under careful monitoring of renal function (II-A).^25^-^27^

### Management of large volume or tense ascites

### Treatment Options available are

- Paracentesis
- Diuretics
- Liver Transplant

### Paracentesis

Paracentesis is a procedure in which a needle or catheter is introduced into the peritoneal cavity to obtain ascitic fluid. It is either done to establish etiology of new-onset ascites or to rule out spontaneous bacterial peritonitis. It is also carried out for therapeutic purpose.^28^-^30^

### Ideal Method of Paracentesis

The left lower quadrant is the best site for a paracentesis. The anterior abdominal wall is thinner 3 cm above and 3 cm medial to the left anterior superior iliac spine. Most of the fluid gravitates at this site in comparison to the midline. This location also best avoids a distended cecum due to lactulose that is commonly used in patients with cirrhosis and the inferior epigastric arteries running in the rectus sheath. A 1.0 or 1.5 inch 20 or 22-gauge needle can be used for paracentesis. Patients with obesity may require a 3.5 inch needle. In those patients where fluid is difficult to localize, ultrasonography can be used for guidance. Paracentesis is best performed by a doctor who is trained for the procedure.^31^ Recently introduced co-axial insertion technique is better than the routine z-technique of inserting cannula for Large Volume Paracentesis (LVP).^32^ LVP is removal of an amount greater than 5L of ascitic fluid and is performed when tense ascites leads to discomfort and respiratory embarrassment or in cases with refractory ascites.^31^ Thrombocytopenia and coagulopathy do not preclude paracentesis FFP transfusion is not needed for correction of deranged INR prior to paracentesis for countering deranged INR.^33^

### Colloid Replacement

LVP ought to be followed by intravenous human albumin infusion to decrease the chances of PPCD. To prevent PPCD, 8g of 20% human albumin is transfused for each liter of ascitic fluid removed when a LVP is performed.^34^,^35^ Half dose albumin,^36^ polygeline,^37^ dextran-70^38^ and normal saline^39^ have been used as alternatives to albumin but without convincing results to show that they hold the same efficacy as albumin (8 gm/L of fluid removed) and are thus not recommended. If less than 5L of ascitic fluid is planned to be removed, albumin infusion can be foregone. Large volume/tense ascites is best managed by LVP followed by diuretic use and a salt restricted diet.

### Safety of LVP

Paracentesis of over 5 litres has been shown to be safe in critically ill patients with tense ascites. It improves respiratory function in such patients including those who are mechanically ventilated with minimal circulatory compromise.^35^,^40^ Large volume paracentesis is better avoided in patients with spontaneous bacterial peritonitis. Some portion of fluid may be removed to bring about relief in symptoms of discomfort or respiratory distress. A few studies have shown that paracentesis under cover of intravenous albumin is feasible in patients with spontaneous bacterial peritonitis and tense ascites without any increase in mortality.^17^,^41^,^42^

Paracentesis is safe in patients with hepatorenal syndrome and tense ascites. In fact, increased intra-abdominal pressure may add to renal compromise. Paracentesis can be performed in such cases with albumin infusion and results in improvement in renal function.^35^ The standard LVP technique consists of placing an 16 G catheter over needle (Intravenous branula) into the peritoneal cavity under aseptic measures, under local anesthesia and draining the fluid into either bags or vacuum containers. The other option is an indwelling peritoneal catheter which can be used to perform paracentesis over several days, as demonstrated in certain trials. Increased risk of bacterial infection of ascitic fluid was noted if the catheter was left for 3 or more days.^43^,^44^

Albumin is known to reduce PPCD but its use after an LVP has not shown any difference in clinical outcomes including encephalopathy, hyponatremia, readmission, renal impairment and death. However in cirrhotic patients with any infection, albumin use may significantly reduce sepsis related mortality and renal impairment.^30^,^45^,^46^

### Recommendations

- Thrombocytopenia and coagulopathy are not contraindications to paracentesis. Platelets or fresh frozen plasma need not be used to correct derangements. (I-A)
- Large volume paracentesis followed by diuretics is appropriate treatment in patients with diuretic sensitive tense ascites (I-A)
- Up to 5 litres of fluid can be removed without colloid support (I-A).
- Large volume paracentesis should be carried out under cover of 8 g of albumin for each litre of ascitic fluid tapped. (I-A).
- Patients must have their discharge weight documented and should be called for first follow up preferably after a week. (I-A)
- Patients must not be subjected to regular paracentesis if adequate weight reduction is achieved with diuretics and salt intake restriction after initial paracentesis. (I-A)
- Fresh frozen plasma (FFP) should not be used as an alternative form of colloid instead of albumin for paracentesis (I-A)
- Paracentesis (with intravenous albumin as colloid support) can be safely instituted in patients with tense ascites and spontaneous bacterial peritonitis (I-A)
- Paracentesis may be safely done in critically ill patients including those on mechanically assisted ventilation (I-B)
- Paracentesis with albumin infusion is safe in patients with hepatorenal syndrome and tense ascites (I-B)
- An indwelling peritoneal catheter can be used to perform paracentesis for up to 3 days (II-C)

## REFRACTORY ASCITES

### Definition and Diagnostic Criteria of Refractory Ascites

Ascites that cannot be mobilized or the early recurrence of which (i.e., after LVP) cannot be satisfactorily prevented by medical therapy.^4^,^47^

### Refractory ascites has two subtypes

### Diuretic-resistant ascites

Ascites that cannot be mobilized or the early recurrence of which cannot be prevented because of a lack of response to sodium restriction and permissible dose of diuretics

### Diuretic-intractable ascites

Ascites that cannot be mobilized or the early recurrence of which cannot be prevented because of the development of diuretic induced complications that preclude the use of an effective diuretic dosage

### Requisites for a diagnosis of refractory ascites:^10^,^14^

1. *Treatment duration:* Patients must be on maximum permissible dose of diuretics i.e. upto spironolactone 400 mg/day and furosemide 160 mg/day for at least 1 week. The patient must adhere to less than 88 mmol/day of sodium in the diet.
2. *Lack of response:* Mean weight loss of less than 0.8 kg over 4 days and urinary sodium output less than the sodium intake.^10^
3. *Early ascites recurrence:* Reappearance of grade 2 or 3 ascites within 4 weeks of initial mobilization.^14^
4. Diuretic-induced complications:
  - Diuretic-induced hepatic encephalopathy is the development of encephalopathy in the absence of any other precipitating factor
  - Diuretic-induced renal impairment is an increase of serum creatinine by >100% to a value >2 mg/dl (177 mmol/L) in patients with ascites responding to treatment
  - Diuretic-induced hyponatremia is defined as a decrease of serum sodium by >10 mmol/L from baseline to a serum sodium of <125 mmol/L
  - Diuretic-induced hypo- or hyperkalemia is defined as a change in serum potassium to <3 mmol/L or >6 mmol/L despite appropriate measures

### Clinical Implications of Refractory Ascites^4^

- Dilutional hyponatremia
- Hepatorenal syndrome
- Spontaneous bacterial peritonitis
- Hepatic hydrothorax
- Spontaneous bacterial empyema
- Umbilical hernia

### Treatment Options for Refractory Ascites

- Large volume paracentesis
- TIPS
- Peritoneovenous shunt
- Liver transplantation
- Vaptans
- Automated Low Flow Ascites (ALFA) Pump

### Large Volume Paracentesis and salt restriction

Serial therapeutic paracenteses are helpful in control of ascites.^12^ If spot urine sodium to potassium ratio is less than one, it suggests a poor response to diuretics. Even in patients with no urine sodium excretion, paracenteses performed fortnightly usually controls ascites.^38^ Diuretics should be stopped if urinary sodium excretion is less than 30 mmol/day^7^

Patients requiring paracenteses of approximately 10 L or more, less than two weeks interval, are likely not following a salt restricted diet.^47^ Repeated therapeutic paracentesis should be reserved for those 10% patients who are proven to be diuretic refractory. Midodrine is as effective as albumin in reducing morbidity and mortality among patients with refractory ascites undergoing LVP at a significantly lower cost. Long-duration midodrine intake can be more useful than shorter duration intake in terms of improvement of renal perfusion and sodium excretion.^48^-^51^ Beta blockers have been proven to have a deleterious effect on survival in patients with refractory ascites and should be stopped.^52^,^53^

### Recommendations

- Serial paracenteses can safely be carried out on outpatient basis for patients with proven refractory ascites every two weeks (I-A)
- Diuretics should be stopped if urinary sodium is less than 30 mmol/day (II-A)
- A salt restricted diet of less than 2g of sodium equivalent to 88 mmol per day must be adhered to even in patients with refractory ascites (I-A)
- Recommendations for colloid replacement and safety of paracentesis remain the same as mentioned for management of tense ascites. (II-A)
- Beta blockers ought to be discontinued in patients with refractory ascites. (II-A)

### Transjugular IntraHepatic Portosystemic Shunt (TIPS)

TIPS is a side-to-side portocaval shunt placed between the portal vein that has high pressure owing to portal hypertension, and hepatic vein with low pressure, to decompress the portal system. It is used in patients with refractory ascites and for patients with variceal hemorrhage that is poorly controlled by endoscopic methods.^54^-^56^

Earlier studies done with non-polytetrafluoroethylene (PTFE) covered stents showed conflicting data as regards patient survival post TIPS. TIPS did show a better control of ascites and better prevention of hepatorenal syndrome. However, encephalopathy was higher in patients undergoing TIPS compared to those undergoing serial paracentesis.^54^-^56^ Newer data suggest a definite advantage in terms of liver transplant free (LTF) survival in patients undergoing TIPS as compared to those undergoing serial paracentesis, with the cost of higher rates of encephalopathy in the TIPS group.^11^

PTFE covered stents work better than uncovered stents in terms of failure rates.^54^ There is some local evidence to suggest that TIPS is a feasible option in patients not suitable for or as a bridge to transplantation in centres where expertise is available.^57^

To undergo TIPS, the patient should not have cardiopulmonary disease and must have normal cardiac ejection fraction (~60%). Patients due to undergo TIPS must have a MELD score of 18 or below.^54^,^56^ Patients with MELD scores between 18 and 24 can undergo TIPS as a bridge to transplant.^58^-^60^ Hence carefully selected patients can undergo TIPS with the risk of higher incidence of post-TIPS heart failure, post TIPS portosystemic encephalopathy without any benefit in overall survival.^58^,^61^

### Recommendations

- TIPS is a feasible option in patients not suitable for or as a bridge to transplantation in centres where expertise is available (I-A).
- It is recommended that patients be evaluated for MELD score and cardiac ejection fraction before undergoing TIPS. (I-B).
- Elective TIPS cannot be encouraged in patients with severe liver failure, concomitant infection, worsening renal failure or cardiopulmonary disease (II-B)
- Only PTFE coated stents are preferred over uncovered stents due to lower shunt failure rates (I-A)
- Regular outpatient paracentesis is a feasible option in patients not suitable for TIPS or liver transplant (I-A)
- Diuretics must not be withheld following TIPS as TIPS may in fact convert diuretic refractory ascites to diuretic sensitive ascites (I-A)

### Peritoneovenous Shunt

A peritoneo-venous shunt is a shunt that drains peritoneal fluid from the peritoneum into either the internal jugular vein or the superior vena cava.^51^

The LeVeen shunt was surgically placed and is not commercially available anymore.^51^

The Denver shunt is a modified from of LeVeen shunt used for management of hydrocephalus. It is placed in a subcutaneous tunnel between the peritoneal cavity and the internal jugular vein by interventional radiologists under local anesthesia.^51^

Potential complications of the peritoneovenous shunt are shunt occlusion, infection, disseminated intravascular coagulation (DIC) manifesting as post-shunt coagulopathy, deep vein thrombosis, catheter breakage, and leaks.^51^

### Recommendations

- Peritoneovenous shunt placement is recommended for patients not suitable for regular outpatient paracentesis, TIPS or liver transplantation, in centres where expertise for performing such procedures is available. (I-A)
- Peritoneovenous shunt placement is not required in diuretic refractory patients who can come for regular outpatient paracentesis. (II-B)

### Liver Transplantation

Liver transplantation is a procedure in which the patient’s diseased liver is replaced by a whole healthy liver (Deceased donor) or part of a healthy liver of a willing donor (Living donor).

The presence of refractory complicated ascites is one of the indications for liver transplantation in patients with decompensated cirrhosis.^38^

### Recommendation

- Since survival after refractory ascites develops is measured in months, such patients should be advised liver transplantation (I-A)

### Vasopressin Receptor Antagonists (Vaptans)

Arginine vasopressin (AVP) has a crucial role in regulation of water and sodium in the body. It acts via three receptor subtypes V1a, V1b, and V2 distributed throughout the body. Vaptans are nonpeptide vasopressin receptor antagonists (VRA). Tolvaptan and Conivaptan are examples.^62^

Due to the ability to cause loss of excess water, the Vaptans have a role in correction of hyponatremia and fluid overload in patients with heart failure and cirrhosis. Some studies recommend the agent Tolvaptan (FDA approved but it is not locally available).^62^-^65^

There are concerns regarding the safety of such agents as regards side effects such as worsening of portal hypertension leading to variceal hemorrhage, hypernatremia, dehydration, deranged renal function, and central pontine demyelination.^66^,^67^

### Recommendations

- Vaptans are newer agents but have potential side effects. The agent Tolvaptan may be used in cirrhotic patients with ascites and hyponatremia but patients need to be carefully monitored for side effects (III-C)
- There is no local evidence on use of vaptans in refractory ascites

### Automated Low Flow Ascites (ALFA) Pump

A surgically implanted pump that automatically transfers peritoneal fluid to the bladder has been undergoing trials for some time (Developed by Sequana Medical AG http://www.alfapump.com/alfapump).

Limited data suggests that it is safe. However long term studies with larger patient populations are ongoing which will further establish main concerns about such a system, especially pump dysfunction (pump failure and bladder and peritoneal catheter related problems), infection, ascitic fluid leakage through wounds, bladder perforation and renal impairment.^68^-^73^

### Recommendations

- Insertion of ALFA pump should only be performed in specialist centres where expertise to place the pump and to deal with its potential complications is available. Pump placement should currently be ideally performed in the setting of a controlled clinical trial (II-C)
- No local evidence is available on results of ALFA pump

### Suggestions for Future Research

- To compare 4 grams of albumin versus 8 grams of albumin per litre of fluid removed during large volume paracentesis (LVP) in prevention of post paracentesis circulatory dysfunction.
- To study the number of patients with cirrhosis and ascites who go on to develop refractory ascites.
- To compare Terlipressin with Albumin versus albumin alone during paracentesis to prevent post paracentesis circulatory dysfunction.
- To diagnose diuretic resistant ascites by the 80 mg IV furosemide challenge test.
- To ascertain benefit of combination of IV albumin and IV furosemide in hypo-albuminemic patients.
- To study the likelihood of spontaneous bacterial peritonitis in patients undergoing large volume paracentesis with use of indwelling catheter.
- To study the efficacy of regular albumin infusions as an adjunct to diuretics for improved survival.
- To study timing and benefits of discontinuation of diuretics in patients with clinically undetectable ascites.
- To study efficacy of eplenerone in patients with cirrhosis and refractory ascites.
- To study the effect of peritoneovenous shunts by interventional radiologists in the local scenario.

### Spontaneous Bacterial Peritonitis: Situation in Pakistan

The incidence of SBP in cirrhotic patients in Pakistan averages 27-38 % of all patients admitted to hospitals, as noted in various studies.^74^-^78^ Up to 10% of asymptomatic patients with cirrhosis presenting for routine outpatient checkups have been found to have SBP.^77^ It was also noted that female gender, history of portosystemic encephalopathy, bilirubin >1 mg/dL and presence of UTI were noted as statistically significant factors among those with recurrent SBP.^79^ A MELD score of >16 corresponds to higher incidence of SBP.^80^,^81^ One study also noted a 34% recurrence rate of SBP in patients who had already experienced an episode of SBP.^76^,^82^,^83^ Zaman A et al & Rajput MR et al observed that low ascitic fluid protein level correlates with development of SBP with high incidence noted in patients with levels lower than 1 g/dL while recent studies state otherwise.^84^-^88^ Causative organisms were seen in the following frequency, E coli 66%, S. pneumoniae 16%, S aureus 8% and Klebsiella 8%.^74^,^77^,^89^ Among rapid diagnostic tools for bedside diagnosis of SBP, the leukocyte esterase dipstick was found to have over 90% sensitivity, specificity, PPV and NPV to diagnose SBP.^90^

One and two-year survival rates following an episode of SBP are 30-40% and 20-30%, respectively.^77^ Once a patient develops SBP, mortality rates of around 30% are reported.^91^

### Recommendations

- Ascitic fluid should be sent for total and differential leukocyte counts, Gram stain and cultures with antibiotic sensitivity in all patients with cirrhosis who are admitted to hospital for any reason, regardless of the interval between current and last admission. This should be done even if recent admission’s ascitic fluid reports have ruled out SBP. This should be done prior to the administration of any antibiotics. Ascitic fluid samples must be transferred to culture bottles at the bedside and transferred for analysis to the laboratory within 30 minutes (I-A)
- Administration of antibiotics to cirrhotic patients immediately following admission should be discouraged and these should only be started once a sample has been obtained for leucocyte counts. (I-A)
- All patients with cirrhosis and ascites presenting with signs of infection such as fever, abdominal pain or encephalopathy should receive empirical antibiotic treatment for possible SBP after ascitic fluid sampling. (I-A)
- Cefotaxime intravenously at a dose of 2g 8 hourly remains the first line antibiotic for all cases of confirmed or suspected SBP.(I-A)
- Ceftriaxone I.V at a dose of 1g 12 hourly is an alternative to the above regime. It is cost effective and has been shown to effectively control SBP. (I-A)
- Switching to a different antibiotic may be done if the patient does not clinically improve and shows a non-significant reduction in leukocyte counts on a repeat ascitic fluid sample obtained 48 hours after the first sample. (I-B)
- Modification to antibiotic regime must preferably be done in light of culture and sensitivity reports. (I-B)
- All patients should receive 1.5 g albumin per kg body weight within 6 hours of diagnostic paracentesis and 1.0 g/kg on day 3. I.V albumin remains very expensive and lower doses of albumin need to be considered in light of new trials. (II-B)
- Proton pump inhibitors are massively over-prescribed (one study quoting a figure of almost 70% patients having no clear indication for PPI use) and they may contribute to the development of SBP in patients with cirrhosis and ascites. PPIs must be used only in patients with clear documented dyspepsia and/or reflux and their use must be short duration as and when symptoms develop. (I-B)
- Secondary bacterial peritonitis must be ruled out in patients with very high leucocyte counts. (I-B)
- In such cases, ascitic fluid must be analysed for protein, glucose, LDH, Gram Stain and CEA along with a contrast enhanced CT of the abdomen & pelvis area to rule-out/rule-in a focal source of infection. (I-B)
- Prophylaxis against SBP must be done in all cases of GI bleeding in patients with liver cirrhosis. (I-A)
- Prophylaxis against SBP in cases of GI bleeding in cirrhotic patients can be effectively done starting with 1g daily of ceftriaxone and switching to oral therapy after endoscopy. (I-B)
- Oral therapy in aforementioned scenario is continued to day 7. (I-B)
- Oral agents that can be used include ciprofloxacin 500mg BID, levofloxacin 500mg OD or norfloxacin 400mg twice daily. (I-A)
- Prophylaxis against SBP is also required for the following two groups:
- Patients who have had one episode of SBP: Such patients require prophylaxis till death or liver transplantation. (I-A)
- Patients with ascitic fluid protein <1.5 g/dL along with renal dysfucntion (creatinine >1.2, BUN > 25 or serum Na < 130) or advanced cirrhosis (Child score 9 and bilirubin 3). Such patients should preferably receive prophylaxis for one year. (I-A)
- Prophylaxis against SBP can be done using once daily doses of ciprofloxacin. It is advisable to use different antibiotics for treatment and prophylaxis in the same patient (II-A)
- Intermittent dosing (such as once weekly) of antibiotics for prophylaxis is not recommended as it may result in emergence of resistant strains of bacteria. (II-C)

### Suggestions for future research

- To study 6-month mortality in patients with and without regular antibiotic prophylaxis against SBP.
- To study the efficacy of ofloxacin and Trimethoprim-Sulfamethoxazole in prophylaxis against SBP.
- To study the difference in yield of TLC/DLC in ascitic fluid collected in vial with EDTA as against a plain syringe in SBP.
- To study the decrease in TLC in ascitic fluid after first dose of antibiotic in patients diagnosed with SBP.

**Table:** 

Baseline sCr	A value of sCr obtained in the previous 3 months when available can be used as baseline sCr. In patients with more than one value, the value closest to the time of admission must be used
Definition of AKI	• Increase in sCr ≥ 0.3mg/dl over 48 hours or
	• A percentage increase in sCr ≥ 50% from baseline which is known or presumed to have occurred within the last 7 days
Staging of AKI	• Stage 1 Increase in sCr ≥0.3mg/dl or an increase in sCr ≥1.5-fold to 2-fold from baseline
	• Stage 2 Increase in sCr >2-fold to 3-fold from baseline
	• Stage 3 Increase of sCr >3-fold from baseline or sCr ≥4.0mg/dl with an acute increase ≥0.3mg/dl or initiation of renal replacement therapy

### Hepatorenal syndrome

Hepatorenal syndrome (HRS) is defined as the occurrence of renal failure in a patient with advanced liver disease, with portal hypertension and ascites, in the absence of an identifiable cause of renal failure.^92^ HRS is thus diagnosed by ruling other possible causes of renal failure.^93^

Two studies carried out in tertiary care hospitals in Sindh, Pakistan, revealed a 15% incidence of hepatorenal syndrome in patients with cirrhosis.^92^,^94^ A similar study carried out in Egypt showed 11.3% incidence.^95^ It is the most common cause of renal impairment in patients with cirrhosis with ascites (47.4%) followed by other causes like primary renal disease, analgesic nephropathy and hypovolemia.^94^ The annual incidence of HRS, as reported in international literature is 7.9%.^93^

### Pathophysiology of hepatorenal syndrome

Proposed mechanisms of HRS are splanchnic vasodilation which is accompanied by renal vasoconstriction.^93^,^96^-^98^ A decreased effective arterial blood pressure also contributes towards decreased renal perfusion.^99^,^100^ There is growing evidence to suggest that systemic inflammation as a result of bacterial infection is another precipitant of Hepatorenal Syndrome-Acute Kidney Injury (HRS-AKI).^101^,^102^ The Lipopolysaccharides released by Gram negative bacteria contribute to increased portal pressure and also promote further decompensation of liver disease.^103^,^104^

### Diagnosis of hepatorenal syndrome

There are two types of HRS. Hepatorenal syndrome type of acute kidney injury (HRS-AKI) formerly Type 1 HRS is a rapidly progressive renal failure in patients with advanced liver failure and is associated with poor prognosis. Hepatorenal syndrome type of chronic kidney disease (HRS type 2) is a slowly progressive renal failure in patients with cirrhosis and refractory ascites.^93^,^96^ Those with HRS have a poor prognosis with average median survival of three months.^105^,^106^ HRS-AKI, if left untreated, can lead to death within a month.^106^

The diagnostic criteria for diagnosis of HRS-AKI have recently been revised by the International ascites club in 2015. The previous definition of an absolute reading of serum creatinine ≥1.5mg/dl for diagnosis of HRS was discarded based on knowledge that patients with cirrhosis have a decreased muscle mass and also have an impaired urea cycle. Moreover, milder derangements in renal function were being missed as a result of this cut-off serum creatinine level. The criteria for urine output was also removed since patients with cirrhosis tend to retain sodium and water as a result of renin-angiotensin-activation-system and tend to be oliguric despite having normal renal function.^107^

The new definitions for AKI in cirrhosis are as follows^108^

There is a retrospective study that diagnosed HRS-AKI on the basis of the current criteria and was able to pick HRS-AKI at stage 1 in 40% of in-hospital patients with cirrhosis and ascites. Those patients who had stage 1 AKI despite a serum creatinine less than 1.5mg/dl still had 3.5-fold 30 day mortality than those without AKI^86^. This further illustrates the importance of picking up even slight rise in serum creatinine level to improve mortality.

## PROGNOSIS

In a study carried out at a tertiary care hospital in Islamabad, mortality was found to be higher in patients with advanced liver disease with renal failure as compared to those without renal failure (31% vs 4.5%). Acute kidney injury was the most common presentation and the most common etiologies of renal dysfucntion were infection and hypovolemia. Reversibility was higher with hypovolemia but greater number of deaths were seen in patients with HRS and sepsis.^109^

### HRS type of AKI (HRS-AKI formerly type 1 HRS)

The HRS-AKI is defined as International club of ascites (ICA) criteria AKI-ICA stage 2 or more while other causes of renal failure have been excluded. Thus other causes of AKI such as hypovolemia, shock, urinary tract obstruction, use of nephrotoxic medication and parenchymal diseases of kidney have been ruled out.^101^,^107^-^109^

The box below gives the revised diagnostic criteria for HRS-AKI

**Table:** 

**HRS-AKI**
• Diagnosis of cirrhosis and ascites
• Diagnosis of AKI according to AKI-ICA
• No response after 2 consecutive days of diuretic withdrawal and plasma expansion with albumin 1g per kg body weight
• Absence of shock
• No current or recent use of nephrotoxic drugs (NSAIDs, aminoglycosides, non-ionic contrast etc)
• No macroscopic signs of structural kidney injury defined as*
► Absence of proteinuria (>500mg/day)
► Absence of microhematuria (>50 RBCs per high power field)
► Normal finding on renal ultrasonography
*Patients who fulfill these criteria may still have structural damage such as tubular damage. Urine biomarkers will become an important tool in differentiating between HRS and acute tubular necrosis

HRS-AKI fails to respond to volume expansion with IV albumin or withdrawal of diuretics^83^. Events such as spontaneous bacterial peritonitis or bleeding from varices cause fluctuation in systemic circulation leading to HRS-AKI.^110^ HRS develops in approximately 30% of patients who develop spontaneous bacterial peritonitis^89^. HRS-AKI can also occur spontaneously but it has also been seen with non-selective beta blockers that also trigger variation in systemic circulation.^105^

There are a few biomarkers that can be utilized for diagnosis of HRS-AKI and make it less of a diagnosis of exclusion. Urinary neutrophil gelatinase-associated lipocalin (NGAL) levels and urinary Interleukin-18 (IL-18) levels have shown a significant difference between each category of AKI.^111^-^113^ Urinary NGAL and IL-18 level is high in ATN; intermediate levels are seen in HRS and low levels in prerenal azotemia. Levels in prerenal azotemia are the same as normal individuals as well as those with stable Chronic Kidney Disease (CKD). Urinary NGAL and urine kidney injury molecule 1 (KIM-1) have been found to be useful to predict HRS in patients with cirrhosis.^114^,^115^ These biomarkers can thus serve as useful tools for diagnosis of and prediction of HRS in cirrhotic patients but their role in clinical practice is yet to be defined. Since there is glomerular tubular reflux, kidneys are not histologically normal in HRS as was previously thought.^116^ These urinary biomarkers will help prevent the need for renal biopsy in cirrhotic patients.

### HRS type of CKD (HRS type 2)

HRS type 2 is a slowly progressive form of renal failure where there is slowly progressive deterioration of renal function in patients who have decompensated cirrhosis and refractory ascites. Patients have oliguria and salt and water retention over the course of several months with a slow but steady decline in renal function. The rest of the criteria remain the same as for HRS-AKI.^92^,^93^ Incidence of HRS type 2 varies and is reported to be between 16 and 61%.^92^,^106^,^117^ Patients with HRS type 2 may ultimately develop HRS-AKI either spontaneously or a precipitating event may be involved.^96^,^117^

Patients with cirrhosis and refractory ascites may have other causes of kidney injury and HRS type 2 is a diagnosis of exclusion. Other etiologies of CKD may complicate HRS type 2. It is therefore difficult to diagnose. The prognosis remains poor, albeit slightly better than HRS-AKI.^110^,^115^,^118^

### Recommendations

- It is important to diagnose AKI in cirrhosis and to identify the possible etiology (I-A)
- There are two types of HRS. HRS-AKI and HRS type 2. (I-A)
- Before a diagnosis of HRS-AKI is reached, other etiologies that must be excluded are hypovolemia, shock, use of nephrotoxic medicines and agents and underlying renal parenchymal disease. (I-A)
- HRS-AKI is diagnosed by noticing even a slight rise in serum creatinine. Careful monitoring of renal function in patients with cirrhosis and ascites is crucial especially if they are hospitalized. (I-A)
- An effort must be made to identify the precipitating cause of HRS-AKI. To name a few, systemic bacterial infections, spontaneous bacterial peritonitis in particular, variceal bleed and use of non-selective beta blockers are common. (II-A)
- An early diagnosis of HRS-AKI is known to decrease mortality (I-II)
- Hallmark of HRS type 2 is a slow but constant decline in renal function and carries a poor prognosis. (I-A)
- Urinary biomarkers may be used to predict HRS-AKI and can be of help in differentiating HRS from other causes of AKI. (II-B)

## MANAGEMENT OF HRS-AKI and HRS TYPE 2

### General Measures

Once diagnosed, HRS-AKI should be treated promptly to prevent further deterioration of renal function. It is important to assess for other complications of cirrhosis and to monitor vital signs, urine output, serum electrolytes, renal and liver function tests. Such patients should ideally be managed in an intensive treatment unit and an effort should be made to measure the central venous pressure.

### Recommendations

- Once HRS-AKI has been established, patients are best managed in the ICU setting with careful monitoring of vital signs and urine output (I-A)
- Diuretics must be stopped and intravenous fluid should be monitored to avoid fluid overload and dilutional hyponatremia. (I-A)
- Once HRS has been diagnosed, a trial of furosemide may be given to maintain urine output in patients who show signs of fluid overload. Spironolactone, however, is best avoided to prevent life threatening hyperkalemia (I-A)
- Since beta blockers have been identified as potential triggers in a recent study, it is better to stop them in patients who might have been taking them earlier as prophylaxis against variceal bleed. (II-B)
- All patients with HRS-AKI should undergo screening for sepsis. Thus blood, urine and ascitic fluid cultures must be sent at first presentation. Antibiotics should be continued even if no signs of bacterial infection are found. (I-A)
- Large volume paracentesis is best avoided in an acute setting but it can be safely carried out to make patients with tense ascites more comfortable. (I-A)

### Specific Therapies

### Vasoconstrictor agents and albumin

Vasoconstrictor agents such as terlipressin, midodrine/octreotide and norepinephrine have been evaluated in combination with albumin for treatment of HRS-AKI. These agents bring about vasoconstriction in the splanchnic vasculature and increase the mean arterial pressure. Albumin is of benefit in patients with sepsis owing to its scavenging, anti-oxidant as well as endothelial stabilizing effect apart from its additional benefit of volume expansion.^117^

Terlipressin is initiated at a dose of 1mg 4 to 6 hourly. Dose can go up to 2mg every 4-6 hours if a reduction of more than 25% is not seen in serum creatinine on day 3. Albumin is started at 1g per kg body weight going upto 100g on day 1 and then continued at 40g/day.^117^,^119^ A dose of 12.5g/day in combination with 0.5 to 1.0 mg Terlipressin 12 hourly has also been found to be effective in a study published from Sind, Pakistan.^120^ A lower dose will be of help in bringing the cost of treatment down. A combination of albumin and terlipressin was shown to be of benefit in 58.3 % patients with HRS-AKI in a setting in Lahore, Pakistan.^121^ Lower serum creatinine at the time of diagnosis of HRS-AKI, absence of hyperkalemia and absence of portal vein thrombosis were seen to predict a better response to therapy.^121^ Terlipressin and albumin have been shown to improve short term survival only.^119^,^120^,^122^ Terlipressin is contraindicated in patients with ischemic heart disease. Patients must be monitored for ischemic events of extremities and splanchnic vasculature while on terlipressin.

A combination of albumin with octreotide at a dose of 200 microgram subcutaneously three times a day and oral at a titrated dose of upto 12.5mg three times a day has been of benefit in a few studies from the US and Europe.^48^,^123^ This combination was found to be superior to dopamine combined with albumin.^124^

Noradrenaline at a dose of 0.5-3mg/h is infused continuously dose is gradually increased to achieve a raise in arterial blood pressure and improvement in renal parameters in HRS-AKI.^125^,^126^ In a meta-analysis that extracted data from four studies only, no difference in reversal of HRS, 30-day mortality or recurrence of HRS was seen between terlipressin and norepinephrine.^127^-^130^ Whereas terlipressin has been associated with cardiovascular and ischemic complications, norepinephrine was shown to have lower incidence of such adverse events. However, the ease of administration of terlipressin as a bolus dose in a peripheral vein in a ward as compared to a continuous infusion of norepinephrine in ICU setting gives a clear advantage to terlipressin despite its higher cost.^128^,^131^

These therapies can also be of short term benefit in patients with HRS-CKD but HRS tends to recur once vasoconstrictor therapy is withdrawn.^106^,^110^,^132^ A response is illustrated by a fall in serum creatinine, and an increase in urine output, arterial pressure and serum sodium levels. A complete response is defined by a fall in serum creatinine to a level within 0.3 mg/dL of baseline according to the International Club of Ascites (ICA).^96^,^122^ If there is no response after 72 hours of treatment, the dose of vasoactive agent should be increased. In those who fail to show an improvement in renal function despite 14 days of treatment, pharmacologic therapy should be discontinued.^93^,^96^,^106^,^122^ Longer treatment durations can be used in patients eligible for liver transplantation who show a response to therapy since it improves outcome after liver transplantation.^61^,^119^,^133^

### Prevention of HRS-AKI

Very few studies are available on prevention of HRS. Pentoxifylline showed some promise at a dose of 400mg three times a day in alcoholic hepatitis.^134^-^136^ Treatment of SBP aggressively also prevents development of HRS.^137^ Norfloxacin at a dose of 400mg per day reduced the incidence of HRS in child class C cirrhosis.^82^

For prevention of HRS-AKI and HRS-CKD, albumin should be administered at a dose of 8g for every litre removed when large volume paracentesis (>5L) is carried out.^35^,^45^,^117^

### Tranjugular intrahepatic portosystemic shunt

TIPS has been of benefit in only a handful of patients with HRS-AKI. A strong statement cannot therefore be made on it being used as an option in HRS-AKI.

It, however, has been of benefit in patients with HRS-CKD in view of improvement in renal function as well as in control of ascites.^55^-^57^,^60^ Patients with type 2 HRS must however be carefully evaluated for cardiac function and MELD before being considered for TIPS. TIPS should not be placed in presence of concomitant infection, progressive renal failure or cardiopulmonary disease.

### Renal replacement therapy

Randomized–controlled trials have not shown a survival benefit of renal replacement therapy (RRT such as hemodialysis) or extracorporeal liver support (ELS) for HRS-AKI and HRS-CKD. Continuous venovenous hemodialysis use may, however, be of some benefit in patients who are hemodynamically unstable.^138^ RRT and ELS such as MARS or Prometheus may be of benefit in patients with HRS-AKI but should be restricted to patients who are candidates for liver transplantation.^139^,^140^ Combined liver and kidney transplantation should be offered to patients on RRT for more than 12 weeks.

### Liver transplantation

Owing to the poor prognosis of HRS-AKI and HRS-CKD, these patients must be referred for liver transplantation as soon as possible.^61^,^133^ Primary graft nonfunction and 30-day mortality rates were higher and 1-, 2-, and 5-year graft and patient survival rates were lower in patients with moderate or severe renal failure.^141^-^144^ It must, however, be emphasized that this poorer outcome in comparison to patients without HRS is due to the fact that renal failure is a poor predictor of outcome of liver transplantation. With HRS contributing towards a higher MELD, patients with HRS should have an early referral for a liver transplantation.^145^,^146^

### Recommendations

### Management of HRS-AKI

- Pharmacologic therapy of terlipressin combined with albumin should be administered in patients with HRS-AKI (I-A)
- Dose of terlipressin can be administered in an incremental manner upto a maximum of 2mg/4 h if there is less than 25% reduction of serum creatinine on day 3 (I-A)
- For those patients who do not respond, treatment should be discontinued within 14 days (I-A)
- Patients on terlipressin must be carefully watched for cardiac dysrrhythmias and for signs of ischemia of extremities and splanchnic vasculature. (I-A)
- Alternatives to terlipressin and albumin are combination of albumin with octreotide and midodrine. Unfortunately, midodrine is not available in Pakistan (II-B)
- A combination of norepinephrine and albumin is also an alternative but very little international and local data is available to support these therapies (II-B)
- There is insufficient data to prove that TIPS can be used in the setting of HRS-AKI not responding to pharmacologic therapy( II-B)
- Renal replacement therapy may be useful only as a bridge to liver transplantation. (II-B)
- HRS-AKI patients must be considered for liver transplantation (I-A)

### Management of HRS type 2

- Terlipressin and albumin is of temporary benefit only in patients with HRS type 2 since HRS tends to recur once therapy is withdrawn (I-A)
- Patients with HRS-CKD should be evaluated for TIPS which might improve renal function and control ascites.(I-A)
- Patients must undergo cardiac evaluation, renal and liver function for MELD before undergoing TIPS (I-A)
- Liver transplantation improves survival in patients with HRS-CKD (I-A)
- Patients with HRS type 2 who require renal support for >12 weeks must be considered for combined kidney and liver transplantation. (I-A) Prevention of HRS
- Pentoxifylline has proven to be of some patients with HRS in the setting of alcoholic hepatitis (II-A)
- Early diagnosis and management of SBP with antibiotics and albumin is known to prevent HRS (I-A)
- Large volume paracentesis is best done under cover of albumin (I-A)

### Suggestions for future research

- To study the incidence of HRS-AKI and HRS-CKD in light of the new International Club Ascites definitions
- To study the efficacy of lower dose of albumin in combination with terlipressin for management of HRS-AKI
- To study the response of combination of albumin and norepinephrine in the setting of HRS-AKI
- To ascertain the utility of urinary biomarkers as predictors of HRS-AKI and its diagnosis
- To study efficacy of TIPS as treatment for HRS-AKI not responding to pharmacologic therapy
- To study efficacy and adverse event profile for TIPS for management of HRS type 2

### Authors Contributions

**BA:** Revised manuscript and gave final approval, **AS:** Wrote a section of manuscript

**AA:** Wrote a section of manuscript, **BFZ:** Revised and did final editing of manuscript, literature search & updated citations and references, **ZA:** Wrote a section of manuscript

**LK:** Wrote a section of manuscript, **JIF:** Wrote a section of manuscript, **MS:** Wrote a section of manuscript, **AAN:** Wrote a section of manuscript, **AAC:** Wrote a section of manuscript, **ZYH:** Wrote a section of manuscript, **MS:** Wrote a section of manuscript.
